# Giant condyloma acuminatum in pregnancy: A case report

**DOI:** 10.1111/dth.12972

**Published:** 2019-07-01

**Authors:** Tao Cui, Jingwen Huang, Bin Lv, Qiang Yao

**Affiliations:** ^1^ Department of Gynecology and Obstetrics West China Second University Hospital, Sichuan University Chengdu Sichuan China; ^2^ Key Laboratory of Birth Defects and Related Diseases of Women and Children (Sichuan University) Ministry of Education Chengdu Sichuan China; ^3^ West China School of Medicine Sichuan University Chengdu Sichuan China

**Keywords:** giant condyloma acuminatum, pregnancy

## Abstract

The giant condyloma acuminatum (GCA), also known as Buschke–Löwenstein tumor (BLT), is a type of human papilloma virus‐associated sexually transmitted infection. Treatment options for condyloma acuminatum remain controversial, but surgery seems to be the best option. The management of GCA during pregnancy is more complicated since one has to evaluate the condition of both the mother and the fetus. In this report, we presented a GCA case in a pregnant woman with giant masses that covered the perineal and perianal region. Considering the gestational age and the fetal neurological risk from the anticipated lengthy procedure of mass removal surgery for tumor of this size, we decided to resect the tumor 2 weeks after the infant was delivered via C‐section.

## 1 INTRODUCTION

The giant condyloma acuminatum (GCA), also known as Buschke–Löwenstein tumor (BLT), a type of sexually transmitted infection, was first described by Abraham Buschke and Ludwig Löwenstein back in 1925 (Buschke & Löwenstein, 1925). GCA is associated with human papilloma virus (HPV) infection, especially types 6 and 11 (Dianzani, Bucci, Pierangeli, Calvieri, & Degener, 1998; Gissmann, deVilliers, & zur Hausen, 1982). Infection risks for GCA are elevated with poor hygiene, local irritation, immunosuppression, HIV infection, multiple sexual partners, and anal intercourse (Kreuter et al., 2010). The most frequently affected areas are perineum, vulva, vagina, perianal region, and rectum (Chu, Vezeridis, Libbey, & Wanebo, 1994). HPV infection in pregnancy has been reported to increase complications during delivery and affect newborns. An emerging body of evidence indicates that vertical transmission of HPV from mother to infant could occur while the infant passes through the infected birth canal or in the case of prematurely ruptured membrane (Hahn et al., 2013; Lee et al., 2013). As thoughts should be given to the appropriate mode of delivery, more patients may require C‐sections (Park et al., 2012). HPV infection should be screened and managed more actively among pregnant women.

GCA involves the development and slow progression of exophytic, ulcerative, and cauliflower‐shaped tumors of significant dimensions that typically infiltrate and invade local tissue without spontaneous resolution. Though lesions do not render distal metastases, their locally aggressive behavior and a relatively high local recurrence complicate the cure process and require more active management (Spinu et al., 2014). In a study with 51 cases, men were found more likely to get infected compared to women (2.7:1) (Trombetta & Place, 2001), and GCA is a rare clinical type of genital wart in the pregnant population, with less than five cases described.

In this report, we presented a GCA case in a pregnant woman with giant masses that covered the perineal and perianal regions and were surgically resected 2 weeks after the infant was delivered.

## 2 CASE PRESENTATION

A 17‐year‐old Chinese female presented to our obstetric clinic with pain in the perineal and perianal area and positional abnormality in the 34th week of gestation. A cord‐like neoplasm about 1 cm × 1 cm in size was found on her perianal region in week 14 of gestation in a private clinic of the remote area where she lived. The patient refused treatment despite explanation of the prognosis by the local doctors back then and administered some topical traditional Chinese medicine herself. However, the patient recounted that the mass kept growing rapidly. Unfortunately, the details of that care encounter were not available. Since she noticed her genital lesion, her husband had presented the same type of neoplasm multiple times, and he cut the cauliflower‐like growth on the penis by himself at home without any further physician visits or follow‐up.

**Figure 1:**
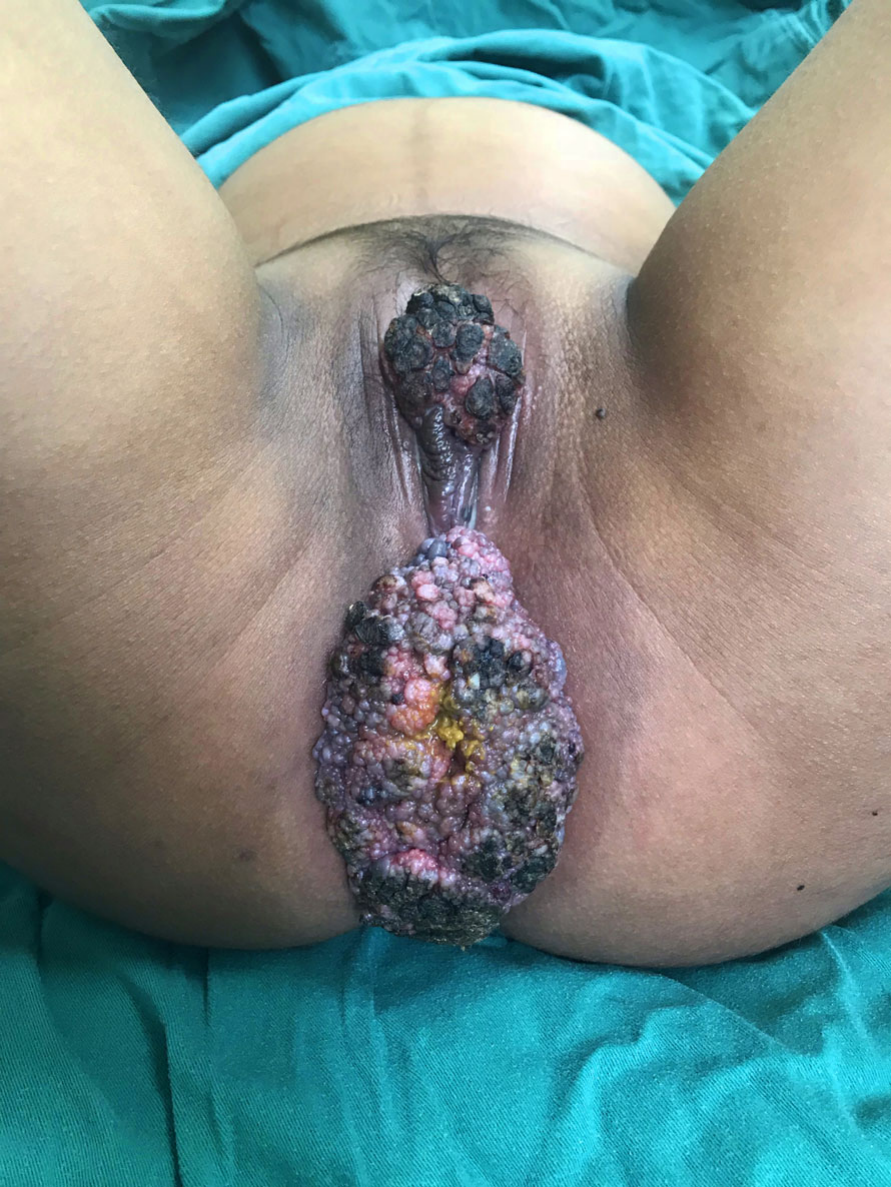
The two large, irregular, ulcerative growths found covering the perineal and perianal area with malodorous discharge on the surface. The anterior one measured 5 cm × 3.8 cm and the posterior one 13 cm × 6 cm

**Figure 2:**
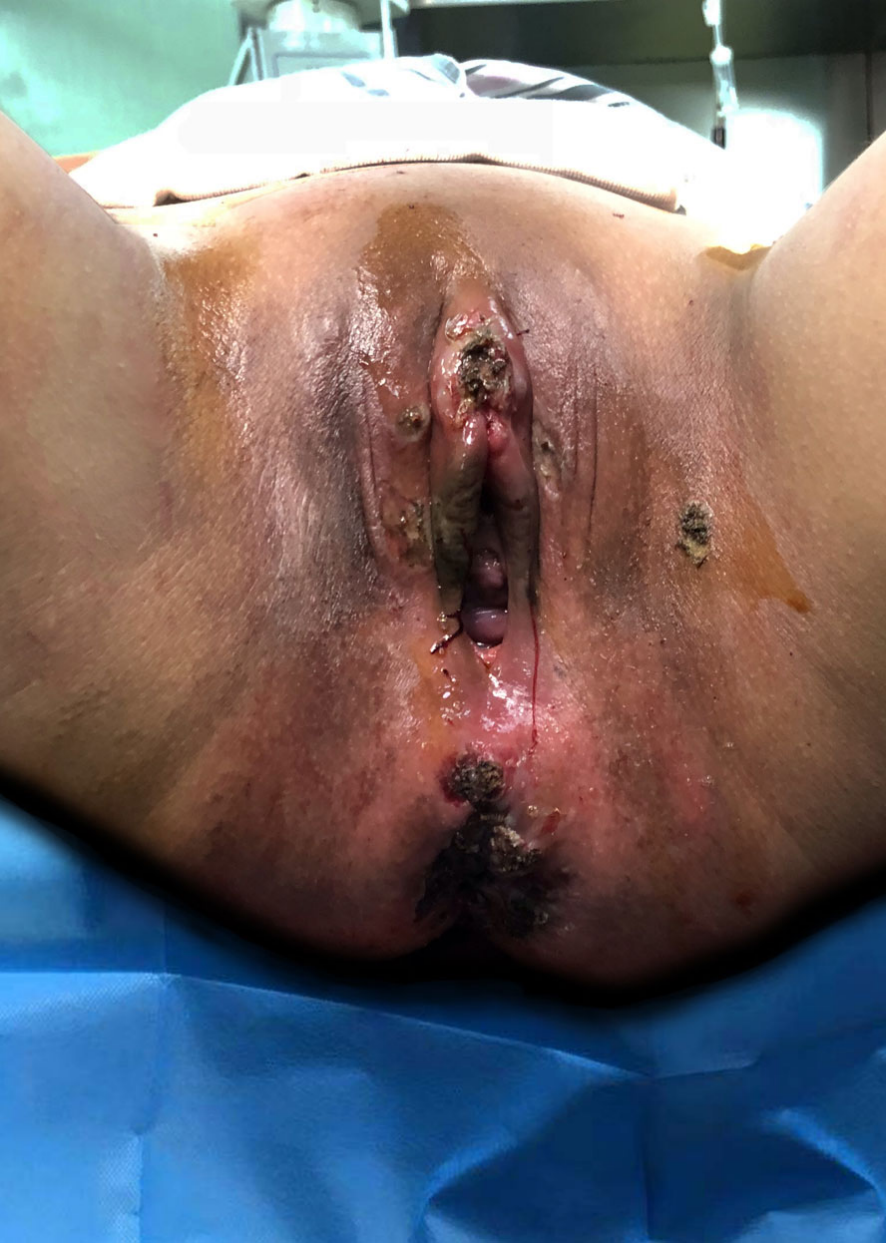
The surgical wound after lesion resection and skin closure

When she presented to our obstetric clinic in week 34 of gestation with perineal pain, she was not able to stand upright as a result of compression from the two large masses. On physical examination, large, irregular, ulcerative, and verrucous vegetations were identified, which covered both the perineal and perianal region with malodorous discharge on the surface (Figure 1). The anterior vegetation measured 5 cm × 3.8 cm and the posterior one 13 cm × 6 cm. We presented the case to the board of multidisciplinary teamwork to discuss the plans for her delivery and the proper sequence of the two procedures, namely the mass removal and the infant delivery. Given the giant vegetation had occupied the vaginal birth canal and that prolonged general anesthesia might increase the neurological risk to the infant, we decided to perform a C‐section before the neoplasm resection. Risks and benefits of infant delivery and resection procedure were discussed with the patient. The infant was delivered successfully via C‐section at full term. Two weeks later, the perineal and perianal giant tumors were excised en bloc at the pedicles by electric scalpel until healthy tissue was reached in gynecologic position under general anesthesia. As the resection did not leave large wound or tissue defect, reconstruction was not indicated for this patient (Figure 2).

**Figure 3:**
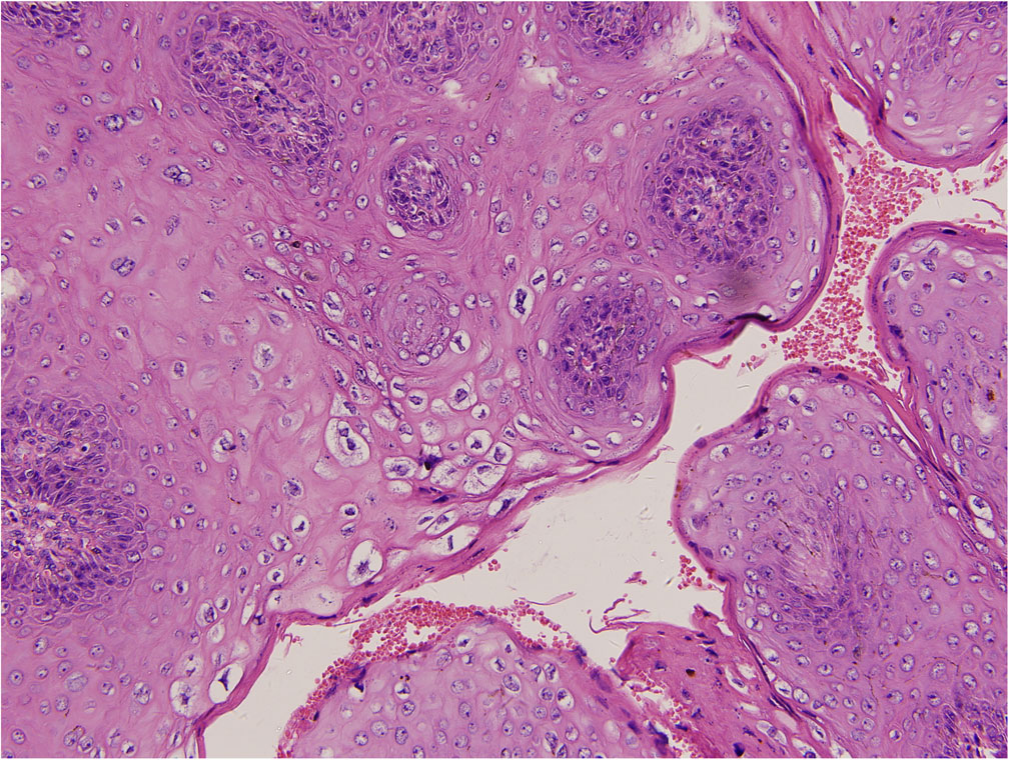
Squamous mucosa with dysplasia and viral cytopathic effect from pathology study of the surgical specimen, consistent with koilocytosis of HPV infection (hematoxylin and eosin staining, ×200)

The resected masses were sent to pathology for staining and sectioning of paraffin‐embedded tissue. The pathological report revealed squamous mucosa with dysplasia and viral cytopathic effect, consistent with koilocytosis in HPV infection (Figure 3). Cytological study on the cervical specimen revealed HPV‐11 positivity, one of the two serotypes associated with GCA from the literature (Dianzani et al., 1998; Gissmann et al., 1982).

After the procedure, the patient was informed of possible recurrence, which is common and presumably related to the infection in the surrounding healthy tissue. Her husband received treatment as well and would be followed up in the local health organization.

## 3 DISCUSSION

The treatment for condyloma acuminata remains controversial, but surgery constitutes the best option. Extended surgical excision or radical local excision with reconstruction of skin has been the primary treatment described (Ahsaini et al., 2013), and minimally invasive surgery has been favored as a first‐line therapy (Spinu et al., 2014). Alternatives reported include CO_2_ laser, electrocoagulation, radiation, intralesional injection of INF‐alpha, or topical imiquimod (Akpadjan et al., 2017; Calderon‐Castrat, Blanco, Santos‐Duran, & Roncero‐Riesco, 2017; Skowron, Raoulx, & Skowron, 2010). Cryotherapy (liquid nitrogen, nitric oxide) associated with topic chemotherapy has been proved effective in patients with small‐sized tumors. Carbon dioxide, argon fluoride, and laser therapy have produced satisfactory results in recurrence treatment. For this case, the giant tumors were excised en bloc by electric scalpel at the pedicles.

The management of GCA during pregnancy is more complicated since clinicians have to evaluate both the mother and the fetus. Vaginal delivery was not an option for this case as the mass had filled up the natural birth canal and increased the risk of vertical transmission of human papillomavirus to the infant as it passes through the infected vagina (Chatzistamatiou, Sotiriadis, & Agorastos, 2016; Park et al., 2012). As it was 34 weeks into her pregnancy, thoughts were given to the appropriate sequence of intervention. The excision of the two giant growths under general anesthesia is a major operation with a high risk of intraoperative maternal bleeding. Nonobstetric surgeries have been shown with risks of spontaneous abortion, intrapartum hemorrhage, premature delivery, low birth weight, etc. (Amos et al., 1996; Jenkins, Mackey, Benzoni, Tolosa, & Sciscione, 2003; Mazze & Kallen, 1989). Therefore, it is generally recommended to postpone elective surgery until after delivery (O'Shea, 2018). Also, the lengthy procedure of GCA resection meant an extended period of anesthesia of the mother, which might delay neurologic development and impair brain function of the fetus. According to a recent alert from the US FDA, fetal exposure to anesthetics in the third trimester of pregnancy amid prolonged surgery may compromise brain development and function, while the association between such exposure within the limited duration, such as epidural anesthesia during C‐section, and learning disabilities has not been established (Olutoye, Baker, Belfort, & Olutoye, 2018). Based on the evidence and the fear of severe and even life‐threatening complications to the patient and the fetus, we decided to deliver the child around the due date through C‐section before we would plan and schedule the GCA resection procedure per the patient's condition, such as her coagulability and the lesion size.

GCA features tendency toward local recurrence and conversion into squamous cell carcinoma (Chao & Gibbs, 2005; Papapanagiotou et al., 2017). The risk of recurrence in the anorectal and perianal regions after excision is 60–66%, with an overall mortality of 20–30%. The development of malignancy has been reported in 30–56% of cases (Akdag & Yildiran, 2018; Indinnimeo et al., 2013). In this case, squamous mucosa with dysplasia and the viral cytopathic effect were noticed, signs of expectable spontaneous regression. Recurrence after an incomplete excision or reinfection to the surrounding tissue remained can be frequent and follow‐up visits for signs of recurrence would be warranted.
